# DeepMolecules: a web server for predicting enzyme and transporter–small molecule interactions

**DOI:** 10.1093/nar/gkaf343

**Published:** 2025-04-29

**Authors:** Alexander Kroll, Yvan Rousset, Thomas Spitzlei, Martin J Lercher

**Affiliations:** Heinrich-Heine-University, Institute for Computer Science and Department of Biology, Universitätsstraße 1, 40225 Düsseldorf, Germany; Heinrich-Heine-University, Institute for Computer Science and Department of Biology, Universitätsstraße 1, 40225 Düsseldorf, Germany; Heinrich-Heine-University, Institute for Computer Science and Department of Biology, Universitätsstraße 1, 40225 Düsseldorf, Germany; Heinrich-Heine-University, Institute for Computer Science and Department of Biology, Universitätsstraße 1, 40225 Düsseldorf, Germany

## Abstract

DeepMolecules is an easily accessible web server for predicting protein–small molecule interactions. It integrates four state-of-the-art models: ESP and SPOT for identifying substrates of enzymes and transporters, respectively, TurNuP for predicting enzyme turnover numbers *k*_cat_, and a model for predicting Michaelis constants *K*_M_. These models use deep learning-generated numerical representations of the proteins and small molecules as input features for gradient-boosted decision tree models, achieving high predictive performance. The web interface accepts protein amino acid sequences and small molecules in SMILES, InChI, or KEGG ID formats, supporting single submissions and batch submissions via Excel files. Beyond its predictive capabilities, DeepMolecules provides a structured interface to experimental data on known interactions and kinetic parameters, offering a comprehensive view of protein–small molecule relationships. Freely accessible at https://www.DeepMolecules.org, the web server supports applications in metabolic engineering, drug discovery, and biocatalyst optimization by identifying potential substrates and quantifying their catalytic properties.

## Graphical Abstract

**Graphical Abstract:**
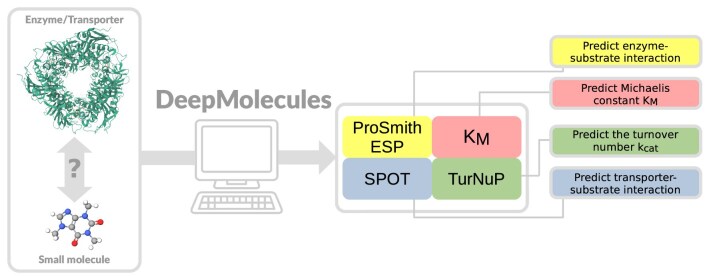

## Introduction

Enzymes and transport proteins are essential biomolecules for maintaining efficient metabolic processes in biological cells. Transport proteins facilitate the movement of molecules across cellular membranes, enabling cells to regulate nutrient uptake, waste removal, and ion balance [1]. Meanwhile, enzymes function as highly specialized biocatalysts that can accelerate chemical reactions by over a million-fold compared to their spontaneous rates [2]. The catalytic efficiency of an enzyme is typically characterized by two kinetic parameters: the Michaelis constant *K*_M_, which reflects substrate binding affinity, and the turnover number *k*_cat_, which quantifies the maximum number of catalytic cycles per active site per second.

Enzymes and transporters often display substrate promiscuity, interacting with secondary substrates in addition to their primary ones [3–5]. A comprehensive understanding of the substrate specificities and kinetics of these proteins is crucial for applications in metabolic modelling, drug discovery, and biotechnology [6]. In pharmaceutical research, insights into substrate binding inform the design of effective enzyme inhibitors [7], while in industry, knowledge of enzyme kinetics and substrate range enables the optimization of biocatalysts for food processing, chemical synthesis, and biofuel generation [8–10]. Identification of transporter substrate specificities can reveal new opportunities in metabolic engineering and drug delivery [11–13].

Despite their importance, the functions of most enzymes and transporters remain poorly characterized, with high-quality substrate annotations available for <1% of all sequenced enzymes and transporters [14]. Even in *Escherichia coli*, arguably the most extensively studied organism, kinetic parameters are known for <30% of its enzymatic reactions [15–17]. This substantial gap in functional and kinetic data limits our ability to fully understand and model metabolic networks. To address this challenge, we have developed the first general models for predicting enzyme and transporter substrates [18–20] as well as the enzyme kinetic parameters *k*_cat_ and *K*_M_ [15, 16].

Here, we introduce DeepMolecules, a user-friendly web server that provides access to these predictive models. The platform features ProSmith, the state-of-the-art enzyme–substrate pair prediction model [20], and SPOT, the first and only general model for identifying substrates of transporters [18]. Both models are binary classifiers that evaluate protein–small molecule pairs and output likelihood scores ranging from 0 to 1, indicating the probability of protein–substrate interactions. Additionally, DeepMolecules provides access to the state-of-the-art models for predicting the kinetic parameters *k*_cat_ [15] and *K*_M_ [16]. Our *k*_cat_ prediction model TurNuP is currently the only model capable of processing complete chemical reaction information to achieve high accuracy. All models are designed for general use, enabling the analysis of any wild-type protein sequence and a wide range of small molecules, without restriction to specific protein families or substrate classes.

DeepMolecules offers significant advancements over the existing web server, combining improved prediction accuracy with broader functionality. Unlike REME [21], the only other enzyme–substrate prediction server, DeepMolecules includes an enhanced version of the original enzyme–substrate prediction model [19], powered by the ProSmith transformer network [20], the first transformer to process protein sequences and potential substrates jointly in the same input sequence. Additionally, DeepMolecules supports batch processing, enabling simultaneous predictions for multiple enzyme–small molecule pairs—a feature missing in REME. It is also the only web server that can simultaneously predict many enzyme kinetic parameters, facilitating the parameterization of genome-scale metabolic models. Finally, DeepMolecules is the first platform to offer a transporter–substrate prediction model.

The web server is designed for ease of use, featuring an intuitive online interface. Users can input protein amino acid sequences through copy and paste, and submit small molecules or chemical reactions as SMILES strings [22], InChI strings [23], or KEGG IDs [24]. DeepMolecules returns not only the prediction results, but also relevant experimental evidence, such as known substrate interactions and kinetic parameters. For high-throughput applications, the platform supports batch submissions via Excel files, allowing users to upload hundreds of entries simultaneously. DeepMolecules is freely accessible to all users, without login requirements, at https://www.DeepMolecules.org.

## Materials and methods

### Underlying data processing and prediction models

**Figure 1.**
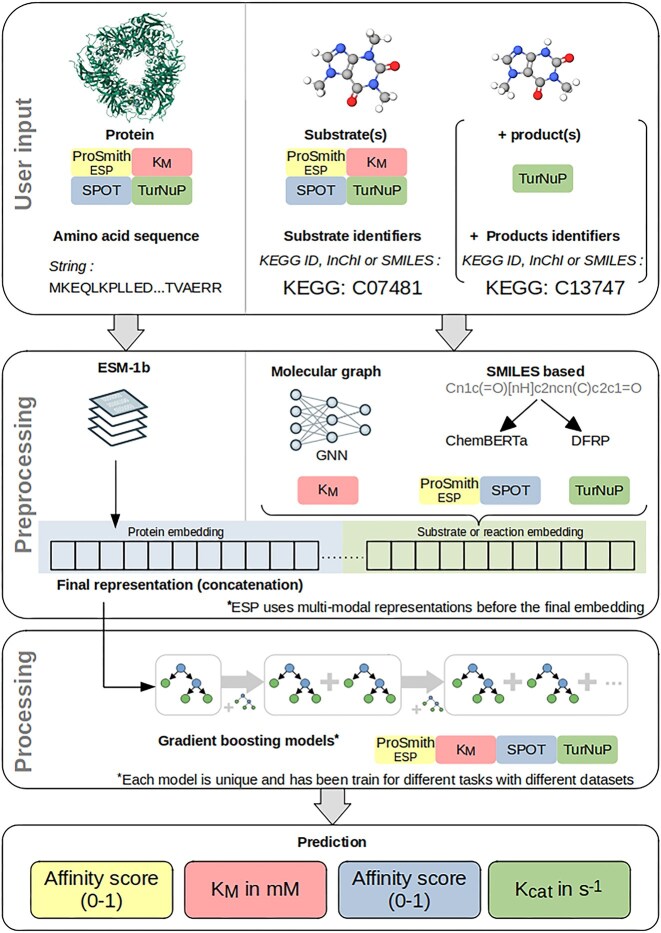
Workflow for processing a single input data point across the underlying models. Each input includes a protein sequence and a substrate identifier; for TurNuP, product identifiers are additionally provided to form a reaction input. The protein sequence is transformed using ESM-1b, while the chemical identifier(s) are mapped to either a Mol file or SMILES strings. The Mol file is used to generate a numerical representation using a graph neural network (GNN), and SMILES strings are numerically encoded using ChemBERTa or DRFP. The resulting chemical representation is concatenated with the protein ESM-1b vector to generate a joint representation of the protein–small molecule pair (or protein–reaction pair for TurNuP). This representation is then entered into a gradient boosting model to produce the final prediction.

Inputs to all DeepMolecules prediction models are protein amino acid sequences and small molecule identifiers (Fig. 1). To apply machine learning techniques, these inputs are converted into numerical representations. Protein sequences are embedded using ESM-1b [25], a transformer-based protein language model pre-trained on masked amino acid prediction tasks. ESM-1b can handle sequences up to 1024 residues; longer sequences are truncated, which may reduce prediction accuracy.

For the enzyme–substrate prediction model ESP, the transporter–substrate prediction model SPOT, and the turnover number prediction model TurNuP, small molecule identifiers are mapped to SMILES strings [22]. ESP and SPOT use ChemBERTa [26], a transformer-based model trained on chemical compounds, to convert SMILES strings into numerical representations. TurNuP receives complete chemical reactions in SMILES format as model input. These chemical reactions are numerically represented by differential reaction fingerprints (DRFPs) [27], which encode the transformations between substrates and products. For the Michaelis constant (*K*_M_) prediction model, small molecules are represented as graphs, and a GNN converts these into numerical representations.

For SPOT, TurNuP, and the *K*_M_ prediction model, the protein and small molecule representations are concatenated into a single joint feature vector. In ESP, protein and small molecule representations are processed by ProSmith [20], a multimodal transformer network that models interactions between proteins and small molecules and then outputs a joint representation. All models use gradient-boosted decision trees [28] to generate predictions from these representations. TurNuP predicts *k*_cat_ values in units of s^−1^, the *K*_M_ model predicts the Michaelis constant in mM, and ESP and SPOT produce likelihood scores between 0 and 1, indicating protein–substrate interaction probability.

The web server uses Django [29], a Python web framework, and is hosted on an institutional server for stable and secure access. Users can submit data through online forms or batch uploads, which are processed by the backend. Once processing is complete, users can retrieve the results through a simple interface (see the ‘Web server workflow’ section).

### Comparison of performance to related models

Until recently, enzyme–substrate prediction models were usually limited to specific enzyme families or substrate classes [30, 31]. ESP [19] was the first general approach, capable of processing any enzyme while outperforming previous, specialized models. In the DeepMolecules web server, we provide access to the newer ProSmith_ESP model [20], which is based on a novel multimodal transformer architecture that significantly improves prediction accuracy. ProSmith_ESP achieves an ROC-AUC (Receiver Operating Characteristic - Area Under the Curve) of 97.2 and an accuracy of 94.2% on independent test data, surpassing ESP’s ROC-AUC of 95.6 and accuracy of 91.5%. Notably, ProSmith_ESP provides reliable predictions for small molecules that are underrepresented in the training set, overcoming a key limitation of previous models.

Earlier approaches for predicting the Michaelis constant *K*_M_ were limited to enzyme–substrate pairs from specific organisms or enzyme families [32, 33], requiring extensive training data for each case, which limited their scalability. Our general *K*_M_ prediction model overcomes these limitations, enabling predictions for any enzyme from any organism. This model uses a GNN to generate representations that capture structural features of the substrate, and a protein language model to numerically encode protein information. The model achieves a coefficient of determination *R*^2^ of 0.53 on an independent test set, representing the current state of the art for predicting *K*_M_ of wild-type enzymes.

Similarly, earlier *k*_cat_ prediction models were limited to specific organisms [17, 32] and required extensive input data, such as reaction fluxes and enzyme active site properties. We developed the general *k*_cat_ prediction model TurNuP, the first general model that outperforms naive similarity-based methods [15, 34]. TurNuP uses the ESM-1b protein language model to encode protein information and is unique in utilizing complete chemical reaction information to provide *k*_cat_ predictions, whereas other models rely on a single substrate. TurNuP achieves a coefficient of determination *R*^2^ of 0.44 on an independent test set and generalizes well to previously unseen enzymes, making it the leading tool for predicting *k*_cat_ values of wild-type enzymes [15].

For transporter–substrate prediction, SPOT is the first general model that predicts specific substrate pairs for any transporter. It outperforms previous support vector machine-based methods like TooT-SC [35] and TranCEP [36], which rely on smaller datasets and predict only broad substrate classes. SPOT uses two transformer networks to generate numerical representations for proteins and substrates, achieving over 92% accuracy on a diverse independent test set. Unlike previous approaches that rely on experimental evidence from similar transporters, SPOT generalizes to unknown proteins from any organism [18].

## Results

### Web server workflow

The DeepMolecules web server provides access to four state-of-the-art models that predict enzyme–substrate pairs (ESP), transporter–substrate pairs (SPOT), turnover numbers *k*_cat_ (TurNuP), and Michaelis constants *K*_M_, respectively. Users can choose between single input or batch input modes. In single input mode, users can enter their data in an online form or use a pre-loaded example. Batch input mode supports Excel (.XLSX) files with up to 500 entries. A downloadable template is available to guide proper formatting. Improperly formatted files trigger an error message, and while the system continues processing valid inputs, predictions are skipped for erroneous entries (e.g. unrecognized identifiers or invalid amino acids).

After submitting a job, the user receives a unique job URL to track progress. The server processes jobs using two queues: CPU-based processing for smaller jobs (≤5 entries) and GPU-accelerated processing for larger jobs, optimizing throughput and minimizing wait times. The server checks for new jobs every 60 s, providing real-time updates on queue status. Completed results are accessible via the unique URL, which remains valid for 48 days before automatic deletion.

**Figure 2.**
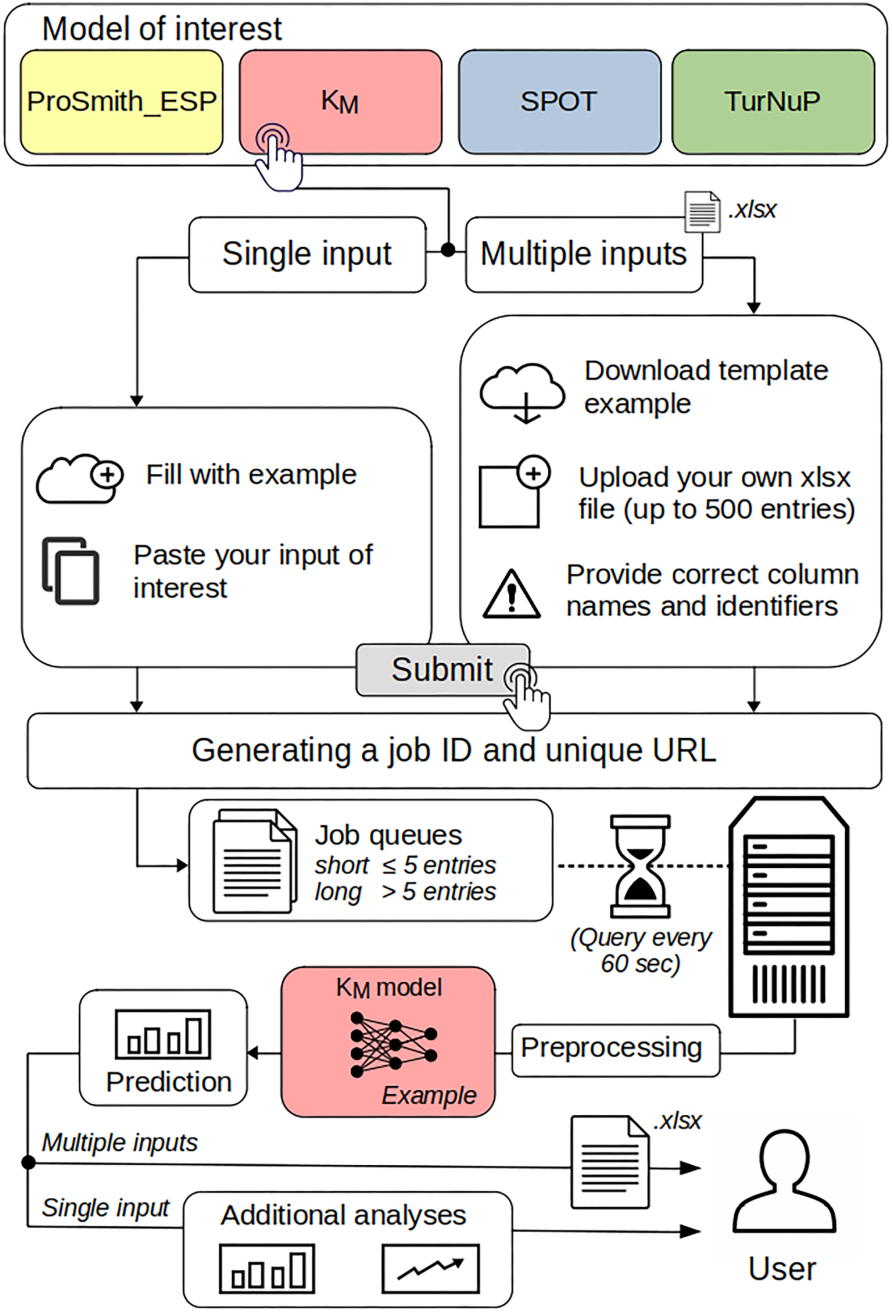
General workflow of the web server for model selection and prediction. This figure illustrates the general workflow of the web server, where users can select from one of four available AI models. After selecting a model, the user is prompted to choose between submitting a single input or multiple inputs. For both options, example inputs are available for download or click, ensuring ease of use. For multiple inputs, users are encouraged to provide correct column names to facilitate proper data processing. After submission, a unique job ID and URL are generated, valid for 48 days. Jobs are queued depending on the number of inputs, either in the short or in the long job queue, with the server querying the database every 60 seconds to check for new jobs. Once the job is started, data preprocessing is performed to format the inputs into the required embeddings for the selected model. The data are then passed through the corresponding gradient boosting model (as illustrated with the *K*_M_ model), and predictions are returned. For single inputs, additional analyses and predictions are provided. For multiple inputs, an updated Excel file with an additional column for the corresponding predictions is returned to the user.

The single input mode offers additional insights (see the ‘Overview of additional analyses’ section). In batch mode, users receive an XLSX file containing all predictions once the job is finished. Figure 2 shows a detailed workflow of the web server process, and a step-by-step tutorial is available on the web server’s help page.

### Interpretation of DeepMolecules’ predictions

DeepMolecules provides clear, interpretable outputs for all prediction types. For enzyme–small molecule and transporter–small molecule classifications (ESP and SPOT, respectively), the server returns prediction scores ranging from 0 to 1. A score above 0.5 indicates that the small molecule is predicted to be a substrate for the protein, with scores close to 1 reflecting high confidence. Conversely, scores close to 0 indicate a low likelihood that the small molecule is a substrate for the entered protein. Scores between 0.3 and 0.7 should be interpreted with caution due to lower prediction accuracy [18, 19]. To aid interpretation, the output also reports the frequency with which the potential substrate appeared in the training data, as predictions tend to be more reliable for small molecules represented at least once as a substrate in the training set [18, 20].

For kinetic parameter predictions (*k*_cat_ and *K*_M_), the server outputs continuous values. Predictions for new enzyme–reaction or enzyme–substrate pairs deviate on average by a factor of 4–5 from the experimentally estimated kinetic parameters consistent with known variability in kinetic data [15, 16]. Similar to the classification models, the output shows how often the substrate or reaction was part of the training set, with accuracy improving when substrates or reactions were present in the training data [15, 16].

### Overview of additional analyses

Beyond basic predictions, DeepMolecules provides comprehensive supporting data, visualizations, and cross-references for all predictions. For enzyme–substrate and transporter–substrate predictions, the server lists experimentally validated substrates for the input protein and known proteins interacting with the queried small molecule. This helps users contextualize predictions within existing biological knowledge. For enzyme–substrate pairs, DeepMolecules additionally computes *k*_cat_ and *K*_M_ predictions for the queried enzyme–metabolite pair.

For the *K*_M_ and *k*_cat_ prediction models, DeepMolecules provides available experimentally measured kinetic parameters from its curated database for the same substrate and/or enzyme as additional data, enabling direct comparisons. All predicted kinetic parameters are visualized alongside the known distribution of experimental kinetic values, helping users gauge the plausibility of the results. Moreover, the platform provides complementary parameters: predicted *K*_M_ values include corresponding *k*_cat_ predictions, and vice versa.

### Use case example

This section describes how DeepMolecules can be used to identify promising substrate candidates for enzymes of unknown function. For this use case example, we selected human cysteine dioxygenase type 1 (CDO1; UniProt ID: Q16878), an enzyme with experimentally validated activity that was not included in our model’s training, validation, or test datasets. To predict potential substrates, we paired the CDO1 protein sequence with all ∼1400 small molecules present in our enzyme–substrate dataset. These small molecules are available for download as an XLSX file from the DeepMolecules homepage. Due to the web server’s input limit of 500 entries per file, the dataset was split into three input files for batch submission.

The predictions yielded 19 small molecules with scores greater than 0.5, suggesting potential enzyme–substrate interactions. Since predictions with scores below 0.7 have lower accuracy and might be unreliable [20], we focused on the five small molecules with prediction scores above this threshold: l-cysteine (prediction score: 0.93), d-cysteine (prediction score: 0.93), alanine (prediction score: 0.93), cysteaminium (prediction score: 0.81), and l-homocysteine (prediction score: 0.80). Results from an experimental validation study [37] confirm l-cysteine as the native substrate of CDO1. While l-homocysteine was predicted as a substrate by DeepMolecules, it was instead shown experimentally to be a weak competitive inhibitor (requiring a 30 000:1 molar ratio to inhibit CDO1 by 50%), suggesting that the model correctly identified its binding potential despite the lack of catalytic conversion. No experimental data are currently available for d-cysteine, alanine, or cysteaminium, but these predictions highlight candidates for further experimental validation.

This use case demonstrates how DeepMolecules can significantly streamline substrate discovery, reducing a pool of 1400 molecules to a manageable list of five high-confidence candidates. By narrowing the search space, DeepMolecules helps focus experimental efforts on the most promising substrate candidates, accelerating the functional characterization of enzymes.

### Limitations

While all implemented prediction models generalize well to proteins that are not highly similar to the training proteins, prediction performance drops significantly if no identical or highly similar substrate was present in the training set. In particular, the transporter–substrate prediction model SPOT was trained with only 364 different substrates, while the enzyme–substrate prediction model was trained with 1379 substrates. Lists of all training substrates can be downloaded for SPOT at https://deepmolecules.org/download_metabolites/metabolites_SPOT and for ESP at https://deepmolecules.org/download_metabolites/metabolites_ESP. Additionally, DeepMolecules reports how often a given substrate appeared during training [15, 18–20], helping users to assess the reliability of individual predictions. Another important consideration is that all models were trained on natural substrates and *in vivo* reactions. As a result, predictions for non-natural substrates or synthetic reactions may be less reliable, as the models have not been exposed to such data during training.

DeepMolecules exclusively models wild-type proteins, as no protein variants or engineered mutants were included in the training data. As a result, the models are not capable of predicting the effects of amino acid substitutions and should be queried only with wild-type proteins.

## Discussion

DeepMolecules provides a comprehensive and user-friendly platform for predicting protein–small molecule interactions and enzyme kinetic parameters. All models provide state-of-the-art prediction accuracy along with additional cross-referenced curated experimental data from BRENDA, Sabio-RK, GO, and UniProt—combining predictive modelling with experimental evidence. The platform stands out by offering generalizable models that are not limited to specific protein families or substrate classes, thereby expanding its applicability across a wide range of biological systems.

Future developments will focus on further improving model performance and expanding functionality. One priority is to enhance predictive accuracy for non-natural substrates and protein mutants, which will likely require new model architectures and the incorporation of corresponding training data. Integrating 3D structural information into molecular representations could also improve the modelling of complex protein–ligand interactions, leading to more precise predictions.

Another important avenue for improvement is the interpretability of model outputs. While DeepMolecules currently provides likelihood scores and confidence metrics, future versions may include explainable AI components that highlight key features driving specific predictions, helping users better understand the underlying biological mechanisms.

Finally, while all prediction models are open source and can be used locally by researchers with programming expertise, the web server was designed to make these powerful tools accessible to a broader audience. By supporting common input formats (SMILES, InChI, and KEGG IDs), providing additional information, and enabling batch processing, DeepMolecules allows users with varying levels of computational expertise to leverage machine learning for biological discovery.

## Data Availability

The DeepMolecules server is freely available at https://deepmolecules.org/.
